# Factors Affecting Onset and Persistence of Metabolic Syndrome in Korean Breast Cancer Survivors: A Prospective Study

**DOI:** 10.3390/ijerph17186814

**Published:** 2020-09-18

**Authors:** Suyoun Maeng, Jungok Yu

**Affiliations:** 1 Department of Nursing, Choonhae College of Health Sciences, Ulsan 44965, Korea; thinknur@ch.ac.kr; 2 College of Nursing, Dong-A University, Busan 49201, Korea

**Keywords:** breast cancer, survivors, metabolic syndrome, onset, cohort studies

## Abstract

This study aimed to investigate the onset and persistence of metabolic syndrome in breast cancer survivors in a community setting. The study included 329 female breast cancer survivors from 39 community health examination centers located in 14 urban areas in Korea. After an average of 4.6 years of follow-up, based on the presence of metabolic syndrome at baseline and follow-up, the subjects were assigned to three groups: Non-metabolic syndrome (*n* = 249), onset (*n* = 32), and persistent (*n* = 48). Factors associated with the metabolic syndrome were analyzed and presented as odds ratios (ORs). Older age, postmenopausal status, lower education, and lower-income level were associated with an increased prevalence of metabolic syndrome in the onset Mets and persistent Mets group. In particular, when the breast cancer survivor was obese (≥25 kg/m^2^), the probability of developing metabolic syndrome was 3.33 times higher than normal-weight subjects (<23 kg/m^2^) and the probability of metabolic syndrome persisting was 16.34 times. When breast cancer survivors were in their 60s or older, the probability of metabolic syndrome persisting was 4.27 times higher than those in their 40s. To prevent the onset and persistence of metabolic syndrome in breast cancer survivors, health-care providers should identify risk factors. Obesity, in particular, should be controlled.

## 1. Introduction

Breast cancer is the most common cancer in Korean women (19.9%) and its incidence increases yearly. The number of people with breast cancer is as high as 198,000 [1]. Despite the high incidence, the relative survival rate of breast cancer has significantly improved to 92.7% [2], due to an increase in cancer screening and a rise in cancer treatment scores. This has resulted in a surge in the number of breast cancer survivors. Breast cancer patients now live longer as cancer survivors [1] and, as a result, are more likely to be exposed to various health issues, such as cardiovascular disease, diabetes, and secondary cancer [3].

In previous studies, breast cancer survivors have been reported to have a 1.3 times higher incidence of cardiovascular disease than the general population [4] and a 1.8 times higher risk of death from cardiovascular disease than women without breast cancer [5]. One study reported that the cause of death for elderly breast cancer survivors was more likely to be cardiovascular disease than the breast cancer itself. Cardiovascular disease, therefore, has a significant impact on the survival and quality of life of breast cancer survivors as they age [6]. Metabolic syndrome is a collection of risk factors for cardiovascular disease, specifically abdominal obesity, hyperlipidemia, diabetes, and high blood pressure [7]. A previous study has shown that the prevalence of the metabolic syndrome in breast cancer survivors was reported to be up to 20–50% [8,9,10], 1.66 times, higher than in those without breast cancer [9].

The high prevalence of metabolic syndrome in breast cancer survivors is associated with the process of cancer treatment. Anticancer drugs and radiation therapy increase the prevalence of hypertension [11]. Antihormone therapy and chemotherapy cause the early onset of menopause [3], increased body fat, and dyslipidemia [12], which have been shown to increase the risk of hyperlipidemia. Breast cancer survivors have also been found to have a higher risk of developing diabetes than the general population two years after the diagnosis [13]. In breast cancer survivors, metabolic syndrome has undesirable effects on the prognosis of breast cancer and the risk of cardiovascular disease, so it is important to identify and prevent risk factors for metabolic syndrome.

Metabolic syndrome is also associated with health behavior. However, it has been reported that the health-promoting behaviors of cancer survivors decrease over time [14]. Most prior studies have compared the risk factors for metabolic syndrome between breast cancer survivors and people without breast cancer in a cross-sectional design [8,9]. However, the risk factors for metabolic syndrome and breast cancer overlap [15], and there is a difference in the risk of metabolic syndrome caused by the process of breast cancer treatment. Therefore, there is a limitation in identifying the risk factors for metabolic syndrome in breast cancer survivors than the general population without breast cancer. Furthermore, it is difficult to identify a causal relationship between risk factors and metabolic syndrome in cross-sectional studies. Therefore, a cohort study for a fixed period is needed to follow patients and examine whether certain factors affect the development of metabolic syndrome and what causes it to persist.

This study used the data of a city-based cohort from the Korean Genome and Epidemiology Study (KOGES) to identify the development of metabolic syndrome in breast cancer survivors and factors related to its persistence. It also aimed to provide basic data for the development of intervention programs to improve the health and quality of life of breast cancer survivors.

## 2. Materials and Methods

### 2.1. Study Population

The city-based cohort study by KOGES of Korea Center for Disease Control and Prevention was a large-scale, community-based genomic survey of adults aged 40–69 who visited a medical institution’s examination center in urban areas. The base investigation was conducted from 2004 to 2013 on 173,209 individuals, and the first follow-up survey of 65,517 people was conducted from 2012 to 2016. In this study, the occurrence and continuation of metabolic syndrome in breast cancer survivors were examined using data from the base investigation and follow-up survey. 

**Figure 1:**
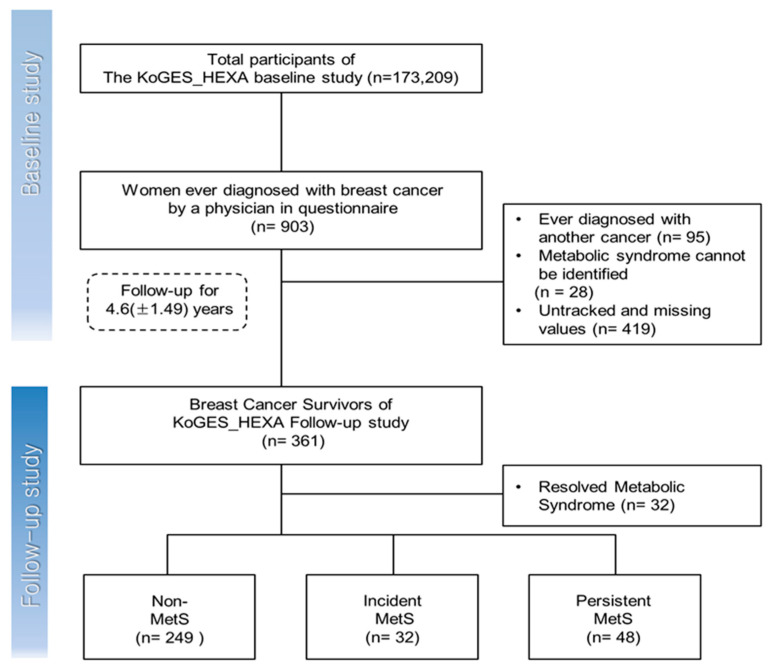
Selection process of subject.

Surviving subjects diagnosed with breast cancer were selected using the definition of the National Convention of Cancer Survivors (NCCS) [16]. Of the 173,209 participants in the base investigation, 903 women were diagnosed with breast cancer by physicians. However, 95 women with a history of other cancers were excluded from the subjects. After excluding the 28 whose metabolic syndrome could not be determined, and 419 who could not be followed up, 361 women were selected for the base investigation. Depending on whether metabolic syndrome had developed, the subjects were divided into three groups. The “no metabolic syndrome group” did not have metabolic syndrome. The “onset metabolic syndrome group” did not have metabolic syndrome at the time of the base investigation but had developed it by the time of the follow-up survey. The “persistent metabolic syndrome group” comprised patients who had metabolic syndrome from the time of the base investigation until the time of the follow-up survey. Thirty-two women who had metabolic syndrome during the base investigation but were not diagnosed at the time of the follow-up survey were excluded from the analysis. Therefore, 329 subjects were selected, with 249 in the no metabolic syndrome group, 32 in the newly developed metabolic syndrome group, and 48 in the continuing metabolic syndrome group. The average duration of follow-up was 4.6 (±1.49) years (Figure 1).

After obtaining research approval from the Korea Center for Disease Control and Prevention, raw data with personally identifiable information removed were obtained and analyzed. The research was conducted with approval from the Dong-A university institutional review board (201908-HR-033-02).

### 2.2. Measurements

#### 2.2.1. Demographic Characteristics

The demographic data included age, level of education, level of income, menopause, and the time of breast cancer diagnosis. A subject was regarded as postmenopausal if she reported no menstruation for the previous 12 months or longer. The level of income was taken as the average monthly income of the family and was classified to below or above the median of 2000 dollars. The time of breast cancer diagnosis was taken as the time that had passed since the diagnosis of breast cancer. This was classified into survival periods of “less than 5 years”, “5–10 years” and “more than 10 years.”

#### 2.2.2. Health Behaviors and Characteristics

The health behaviors and characteristics examined were alcohol consumption, eating habits, physical activities, stress, and body mass index (BMI). Smoking was not included as a variable, since only three (0.9%) out of 329 subjects were smokers. Alcohol consumption was categorized into nondrinking, past drinking, and currently drinking. For eating habits, based on the research by Park et al. [8], the level of carbohydrate intake was analyzed after classification into three categories of low (0–25%), medium (25–75%), and high (75–100%) intake, depending on the consumption in comparison to the total food intake per day. Physical activity was analyzed by dividing the subjects according to their answers (yes or no) to the question, “do you regularly exercise to the point of sweating?”. For stress evaluation, the Psychological Wellbeing Index-Short Form (PWI-SF) [17] was used, which consists of a four-point scale (3-2-1-0) representing “always,” “often,” “somewhat,” and “not at all.” The stress level is measured by summing up the total score, and a high score indicates high stress levels. A total score of 0–8 is classified as healthy, 9–26 as potential stress, and 27 and over as high-risk stress. We divided subjects into high-risk and non-high-risk for analysis, using 27 points as the base. Body mass index (BMI, kg/m^2^) was calculated using height (cm) and weight (kg) and was analyzed by categorizing the participants into normal weight or below (<23 kg/m^2^), overweight (23–24.9 kg/m^2^), and obese (≥25 kg/m^2^) according to World Health Organization’s standard for Asian populations.

#### 2.2.3. Metabolic Syndrome

The current metabolic syndrome criteria were based on the outcomes of a meeting between several major organizations attempting to unify their criteria [18]. However, a World Health Organization report recommends using waist circumference values of ≥90 cm for Asian men and ≥80 cm for Asian women [19]. Thus, for this study, the criteria for diagnosing metabolic syndrome were as follows: (1) Waist circumference of ≥90 cm for men and ≥80 cm for women, (2) triglyceride levels of ≥150 mg/dL, (3) High density lipoprotein cholesterol levels of <40 mg/dL for men and <50 mg/dL for women, (4) blood pressure of ≥130/85 mmHg, and (5) fasting blood glucose levels of ≥100 mg/L.

### 2.3. Data Analysis

Breast cancer survivors were divided into the no metabolic syndrome group, onset metabolic syndrome group, and persistent metabolic syndrome group. The general characteristics, health behaviors, and metabolic syndrome indicators were analyzed with frequency, percentage, mean, and standard deviation and were compared using the ANOVA and chi-square tests. In addition, the odds ratio and 95% confidence intervals were calculated using multinomial logistic regression to determine the effects on the development and continuation of metabolic syndrome of each variable. IBM SPSS Statistics package, version 24.0 (IBM Co., Armonk, NY, USA) was used for data analysis, and significance was set to *p* < 0.05.

## 3. Results

### 3.1. Comparison of the Demographic Characteristics

A total of 329 women participated in the study, with 249 (75.7%) having no metabolic syndrome, 32 (9.7%) having newly developed metabolic syndrome, and 48 (14.6%) having sustained metabolic syndrome. The average age of the subjects was 52.8 (±6.82) for the no metabolic syndrome group, 54.8 (±6.22) for the onset metabolic syndrome group, and 57.5 (±6.87) for the persistent metabolic syndrome group. The rate of menopause was higher in the onset (81.3%) and persistent (95.8%) metabolic syndrome groups than in the no metabolic syndrome group (78.7%). High rates of those who were high school graduates or lower were observed in the onset (84.4%) and persistent (89.4%) metabolic syndrome groups. Higher percentages of those with an average monthly income of less than $2000 were observed in the onset (46.9%) and persistent (45.8%) metabolic syndrome groups than in the no metabolic syndrome group (30.1%) (Table 1).

### 3.2. Comparison of Health Behaviors and Characteristics

The health behaviors did not show statistically significant differences between the three groups. BMI showed a clear difference, however, with the no metabolic syndrome group corresponding to the healthy category with an average of 22.66 (±2.37) kg/m^2^, onset metabolic syndrome group to the overweight category with an average of 24.31 (±2.75) kg/m^2^, and persistent metabolic syndrome group to the obese category with an average of 26.61 (± 3.75) kg/m^2^. The rate of obesity above 25 kg/m^2^ was higher in the onset (34.4%) and persistent (64.6%) metabolic syndrome groups than in the no metabolic syndrome group (14.1%). In the case of carbohydrate intake, there was no subject in the low (0–25%) intake group. The onset and persistent metabolic syndrome groups showed higher carbohydrate intake than the no metabolic syndrome group. On the stress score, the rate of high risk with 27 points and above was higher in the onset and persistent metabolic syndrome groups, but without statistical significance (Table 2).

### 3.3. Factors Influencing Metabolic Syndrome

Multinomial logistic regression analysis was performed to identify the factors that influence metabolic syndrome, using age, menopause, level of education, level of income, and BMI variables, which were the significant variables from the univariate analyses. BMI was the factor that affected the new onset of metabolic syndrome, with obese participants (≥25 kg/m^2^) being 3.33 times more likely to develop it than those who were of normal weight or lighter. The factors that affected the persistence of metabolic syndrome were age and BMI. The probability of sustaining metabolic syndrome was 4.27 times higher in breast cancer survivors who were in their 60s and older than those in their 40s and 16.34 times higher for those who were obese than people of normal weight (Table 3).

## 4. Discussion

In this study, the city-based cohort data of KOGES were used to identify factors contributing to the development and persistence of metabolic syndrome in breast cancer survivors. Of the 329 subjects, 80 were affected by metabolic syndrome at the time of the follow-up survey, accounting for 24.3%. This was consistent with the prevalence of 20–50% [8,9,10] in previous studies and with the prevalence of metabolic syndrome (26.8%) in existing breast cancer survivors in Korea [8]. Of the 80 breast cancer survivors, 32 did not have metabolic syndrome at the time of the base investigation but had developed metabolic syndrome by the time of the follow-up survey. From the time of the base investigation to the time of the follow-up survey, 48 patients had sustained metabolic syndrome for 4.6 (±1.49) years. The recurrence and mortality rates of survivors with metabolic syndrome are reported to be 1.65 times higher than those without [20]. The quality of life related to health is lower in women with persistent metabolic syndrome than in those without [21]. Therefore, health-care providers should make efforts not only to determine the occurrence of metabolic syndrome in breast cancer patients but also to review and manage the syndrome.

The univariate analyses showed that the variables affecting the development and continuation of metabolic syndrome in breast cancer survivors were age, menopause, level of education, level of income, and BMI. The ratio of people in their 40s was low and for age 60 and older was high in the onset and persistent metabolic syndrome groups than the no metabolic syndrome group. This is consistent with a previous study that increased age of cancer survivors increases the risk of metabolic syndrome [22]. Furthermore, the percentage of menopausal subjects in the onset and persistent metabolic syndrome groups were found to be high, with that of continuing metabolic syndrome group being particularly high at 95.8%. This is similar to a study [9] of postmenopausal women that reported the risk of metabolic syndrome to be higher in breast cancer survivors than women who had not had breast cancer. Our results confirm that it is necessary to identify and actively manage the risk factors for metabolic syndrome in breast cancer survivors in the community. There is an increased risk of development and continuation of metabolic syndrome if a survivor is of older age or postmenopausal.

Our analysis of socioeconomic status showed that the onset and persistent metabolic syndrome groups had lower levels of education and income than the no metabolic syndrome group. This is consistent with the report of a previous cohort study [23] showing an increased risk of metabolic syndrome based on the levels of education and income in Korean women in general. Breast cancer survivors also differ in their risk of metabolic syndrome, depending on their socioeconomic status. Income level affects lifestyle, which further influences metabolic syndrome. A prior study found that the lower the income, the higher the percentage of smokers and the lower the level of exercise [24]. The lower the level of income, the more likely was the consumption of low-cost, high-caloric food [25]. These findings suggest that there is a need for the development of a differentiated strategy for the prevention and management of metabolic syndrome in low-income breast cancer survivors in the community and a need for a national policy and institutional intervention. It also shows that, for accurate understanding, further follow-up surveys focused on levels of income should be conducted to identify the factors that influence health.

Obese breast cancer survivors were 3.33 times more likely to develop metabolic syndrome and 16.34 times more likely to sustain it than those of, or below, normal weight. According to a previous study, obesity not only causes metabolic syndrome [26] but also has direct effects on cardiovascular disease [27]. Furthermore, it is reported that obesity increases the cancer recurrence rate and side effects in breast cancer survivors [28]. It reduces survival rates, resulting in a 1.81 (95% CI 1.42–2.32) times higher mortality rate than those of normal weight [29]. As such, obesity in breast cancer survivors is important for the prognosis and, therefore, weight control is essential for breast cancer survivors.

Interventions for exercise and diet in the obesity management of breast cancer survivors are known to be effective [30]. In this study, there was no significant association of health behavior, including exercise and diet, with the development and continuation of metabolic syndrome. However, intervention in exercise and dietary control should be provided. In the case of physical activity in this study, all three groups had similar regularity. This may be because the survey was not specific, which could have hindered the analysis of the details. Therefore, additional research is necessary to supplement the questions related to exercise. 

With regard to carbohydrate intake, all three groups of breast cancer survivors were consuming more than the appropriate intake for Koreans of 55–65% [31]. Carbohydrate consumption was slightly higher in the onset and persistent metabolic syndrome groups than in the no metabolic syndrome group. According to studies by Park et al., breast cancer survivors had 2.48 times higher risk of metabolic syndrome if they consumed excessive amounts of carbohydrate than those who did not have breast cancer [8]. Therefore, more studies of carbohydrate intake and metabolic syndrome among breast cancer survivors are needed in the future.

This study had some limitations. First, it was impossible to control the effects of breast cancer treatment on metabolic syndrome because the research was conducted through secondary data and the details of the treatment process each individual underwent were unknown. The subjects of this study may have been in better health as they were able to visit the local health examination center. The possibility of those who could not be followed being admitted to a hospital due to worsening condition or death cannot be ruled out.

Despite these limitations, this study is meaningful in that it used cohort data, based on an average of 4.6 years of follow-up, to reveal factors that affect the development and continuation of metabolic syndrome and discussing the findings in terms of public health care.

## 5. Conclusions

This study identified risk factors for the onset and persistence of metabolic syndrome in breast cancer survivors. Older patients, those who were postmenopausal, who had lower levels of education and income, and the more obese were more likely to develop or have persistent metabolic syndrome. Based on these findings, it is important for health-care providers to identify the risk factors associated with breast cancer survivors in the community and to mediate them in a multifaceted manner. Patients who are obese or who are in their 60s or older may continue to have metabolic syndrome. Furthermore, differentiated strategic interventions should be prioritized for those with lower levels of education and income, as metabolic syndrome and the socioeconomic level of breast cancer survivors are related. 

The proper management of metabolic syndrome based on the characteristics of breast cancer survivors in the community reduces the incidence and risk of cardiovascular disease. It is of great importance in improving the quality of life of breast cancer survivors and ultimately reducing unnecessary social costs for health care in the community. Based on the results of this study, it is necessary to develop and implement a multifaceted mediation management program for the management of obesity. Further cohort and extended studies for breast cancer survivors are necessary, as breast cancer survivors are at high risk of developing cardiovascular disease as a result of the development and persistence of metabolic syndrome.

## Figures and Tables

**Table 1:** Sociodemographic characteristics among breast cancer survivors.

Variables	Non-MetS (*n* = 249) M ± SD or *n* (%)	Onset MetS (*n* = 32) M ± SD or *n* (%)	Persistent MetS (*n* = 48) M ± SD or *n* (%)	F or χ^2^	*p*
**Age (yr)**	**52.8 ± 6.82**	**54.8 ± 6.22**	**57.5 ± 6.87**	**11.31**	**<001**
40–49	81 (87.1)	7 (7.5)	5 (5.4)	21.36	<001
50–59	129 (75.9)	18 (10.6)	23 (13.5)		
≥60	39 (59.1)	7 (10.6)	20 (30.3)		
Menopausal status					
Postmenopause	196 (73.1)	26 (9.7)	46 (17.2)	7.81	0.020
Premenopause	53 (86.9)	6 (9.8)	2 (3.3)		
Education (yr)					
≤12	166 (70.6)	27 (11.5)	42 (17.9)	8.75	0.013
>12	68 (87.2)	5 (6.4)	5 (6.4)		
Income ($)					
<2000	75 (67.0)	15 (13.4)	22 (19.6)	7.03	0.030
≥2000	174 (80.2)	17 (7.8)	26 (12.0)		
After diagnosis (yr)					
<5	132 (79.0)	17 (10.2)	18 (10.8)	5.23	0.264
5–9	67 (76.1)	7 (8.0)	14 (15.9)		
≥10	48 (67.6)	8 (11.3)	15 (21.1)		

**Table 2:** Health behavior characteristics among breast cancer survivors.

Variables	Non-MetS M ± SD or *n* (%)	Onset MetS M ± SD or *n* (%)	Persistent MetS M ± SD or *n* (%)	χ^2^	*p*
Drinking status					
Never or Past	212 (75.7)	24 (8.6)	44 (15.7)	4.28	0.118
Current	36 (75.0)	8 (16.7)	4 (8.3)		
Exercise					
Regularly	116 (74.8)	15 (9.7)	24 (15.5)	0.19	0.910
Irregularly	133 (76.4)	17 (9.8)	24 (13.8)		
Carbohydrate intake					
25–75%	129 (78.2)	13 (7.9)	23 (13.9)	1.40	0.497
75–100%	117 (73.1)	18 (11.3)	25 (15.6)		
Stress score					
<27	217 (76.7)	26 (9.2)	40 (14.1)	1.50	0.472
≥27	25 (67.6)	5 (13.5)	7 (18.9)		
Body mass index	22.66 ± 2.37	24.31 ± 2.75	26.61 ± 3.75	47.06	<001
<23 kg/m^2^	153 (89.0)	12 (7.0)	7 (4.0)	64.54	<001
23–24.9 kg/m^2^	61 (76.3)	9 (11.2)	10 (12.5)		
≥25 kg/m^2^	35 (45.5)	11 (14.3)	31 (40.2)		

**Table 3:** Multivariate odds ratios for metabolic syndrome in breast cancer survivors.

Variables	Onset MetS		Persistent MetS	
	aOR (95% CI)	*p*	aOR (95% CI)	*p*
Age (yr)				
≥60	1.57 (0.43–5.75)	0.491	4.27 (1.10–16.62)	0.037
50–59	1.44 (0.49–4.23)	0.505	1.67 (0.47–6.00)	0.428
40–49	1		1	
Menopausal status				
Postmenopause	0.87 (0.28–2.70)	0.815	4.03 (0.77–21.09)	0.098
Premenopause	1		1	
Education (yr)				
>12	0.64 (0.22–1.81)	0.397	0.42 (0.13–1.38)	0.153
≤12	1		1	
Income ($)				
<2000	1.45 (0.65–3.26)	0.364	0.96 (0.44–2.06)	0.911
≥2000	1		1	
Body mass index				
≥25 kg/m^2^	3.33 (1.32–8.43)	0.011	16.34 (6.35–42.06)	<001
23–24.9 kg/m^2^	1.66 (0.66–4.19)	0.284	2.61 (0.90–7.53)	0.077
<23 kg/m^2^	1		1	
